# Damage severity of wood-destroying insects according to the Bevan damage classification system in log depots of Northwest Turkey

**DOI:** 10.1038/s41598-020-70696-6

**Published:** 2020-08-13

**Authors:** Mesut Yalcin, Caglar Akcay, Cihat Tascioglu, Besir Yuksel, Ali Kemal Ozbayram

**Affiliations:** 1 Department of Forest Products Engineering, Faculty of Forestry, Duzce University, 81620 Duzce, Turkey; 2 Department of Forest Engineering, Faculty of Forestry, Duzce University, 81600 Duzce, Turkey

**Keywords:** Forest ecology, Forestry, Invasive species

## Abstract

The aim of the study was to determine damage severity of wood-destroying insects on logs stored in forest depots. The Bevan damage classification (BDC) system, developed in 1987, was utilized to determine damage severity in log depots in 21 locations throughout seven provinces in Turkey. Pheromone traps were placed in those locations at the beginning of April in 2015 and 2016. Furthermore some stored wood within the log depots were checked and split into small pieces to collect insects that damage wood. The BDC system was used for the first time to measure the severity of insect damage in log depots. Twenty-eight families, 104 genera and 123 species were identified in this study. Based on the BDC system, the highest damage was found from the Cerambycidae and Buprestidae families. *Arhopalus rusticus* was determined as the insect responsible for the highest amount of damage with 8.8% severity rating in the pheromone-trapped insects group. When the stored wood material was considered, *Hylotrupes bajulus* was found to be the cause of the highest damage. The lowest damage values were among the predator insects (Cleridae, Trogossitidae, Cantharidae) and those feeding on fungi colonized on the wood (Mordellidae, Cerylonidae, Nitidulidae). Some other predator insects of the Tenebrionidae family (*Uloma cypraea, Uloma culinaris, Menephilus cylindricus*) and Elateridae family (*Lacon punctatus*, *Ampedus* sp.) exhibited relatively higher damage severity values since they had built tunnels and made holes in the stored wood material. When the environmental factors were considered, the Buprestidae family exhibited a very strong positive relationship (p < 0.005) with insect frequency distribution (r = 0.922), number of species (r = 0.879) and insect density (r = 0.942). Both families showed the highest number and frequency during July and August, highlighting the importance of insect control and management during these months.

## Introduction

Insects are one of the most important biological factors that destroy wood materials^1^. Insects from the Cerambycidae, Anobiidae, Lyctidae, Buprestidae and Melandryidae families cause damage to logs and lumber that lowers technical specifications, leading to major economic losses^2^. The Tenebrionidae, Elateridae, Lucanidae and Scarabaeidae families, on the other hand, produce their larvae on long-term stored wood and partially decayed wood material^3^. The Cleridae and Trogossitidae families are common since they are predators of wood-destroying insects^4–6^. Insects from the Curculionidae/Scolytinae families destroy wood at every stage from stump to log^7^.

Insects from the Cerambycidae family cause damage on both softwood and hardwood species. Their major destruction takes place during the larval stage^8^. Some species of Cerambycidae damage ornamental and fruit trees, while other species harm newly harvested trees, inner bark, decaying wood, dried wood and furniture in use^9–11^. Some species are considered invasive for some locations, making protective and control measures very difficult^12^. They top the list among insects that are economically harmful for wood material^13^.

Insects from the Buprestidae family can survive under high temperatures that other insects cannot tolerate^14^. Adults do not cause major damage to flowers. Their larvae, however, heavily attack the bark, cambium and wood portions of soft and hardwoods^9,15^. They exhibit high reproduction rates on partially burned trees after forest fires. Generally, they are considered as secondary harmful insects; however, if they cannot find a proper food source, they became primary harmful insects. The Buprestidae family is an important family with a high number of species creating major damage on forest and wood products^16^.

The Anobiidae family of insects can damage both soft and hardwoods. They generally attack sapwood, and occasionally hardwood. They can turn construction wood into a sponge-like material, thus causing heavy damage. The most important species of this family are *Anobium punctatum* (furniture beetle), *Xestobium rufovillosum* (deathwatch beetle)^17^, *Ptilinus pectinicornis* (fan-bearing wood-borer) and *Ernobius mollis* (pine bark anobiid or pine knot borer)^18^.

Scolytinae (Curculionidae) (bark beetles) are secondary harmful insects that prefer trees weakened due to wind and snow load, forest fires, drought or infestation by other insects^19^. Even though they are considered secondary harmful insects, they tend to turn into primary harmful insects during their maturation into young adults by attacking healthy trees. The bark beetles harm bark, outer sapwood and cambium zones. Ambrosia beetles are an important member of this subfamily and cause degradation via fungus transfer^20^.

Insects from the Elateridae and Tenebrionidae families live in decaying wood material and dead or living vegetation in forested areas and are known as predators^21^.

Log depots are storage facilities for logs harvested from the forest until they are sent to the final consumers. During this storage time, the wood is attacked by insects, resulting in major economic losses^22,23^. In order to manage these harmful insects and reduce the scale of damage to the wood, identification of the insect species in the storage areas must be carried out. Most previous studies have focused on insects causing damage to living trees rather than on harvested material held in outdoor storage. Therefore, the current study aimed to investigate and identify the insect species causing economical loss to industrial wood material in log depots. Insects were collected via a unique pheromone trap. Additionally, the Bevan damage classification system was utilized for the first time to calculate economic losses. The insect frequency, insect number and species number of wood-destroying insects based on provincial locations were determined and their relation with the environmental factors was discussed.

## Material and methods

### Study areas

**Figure 1:**
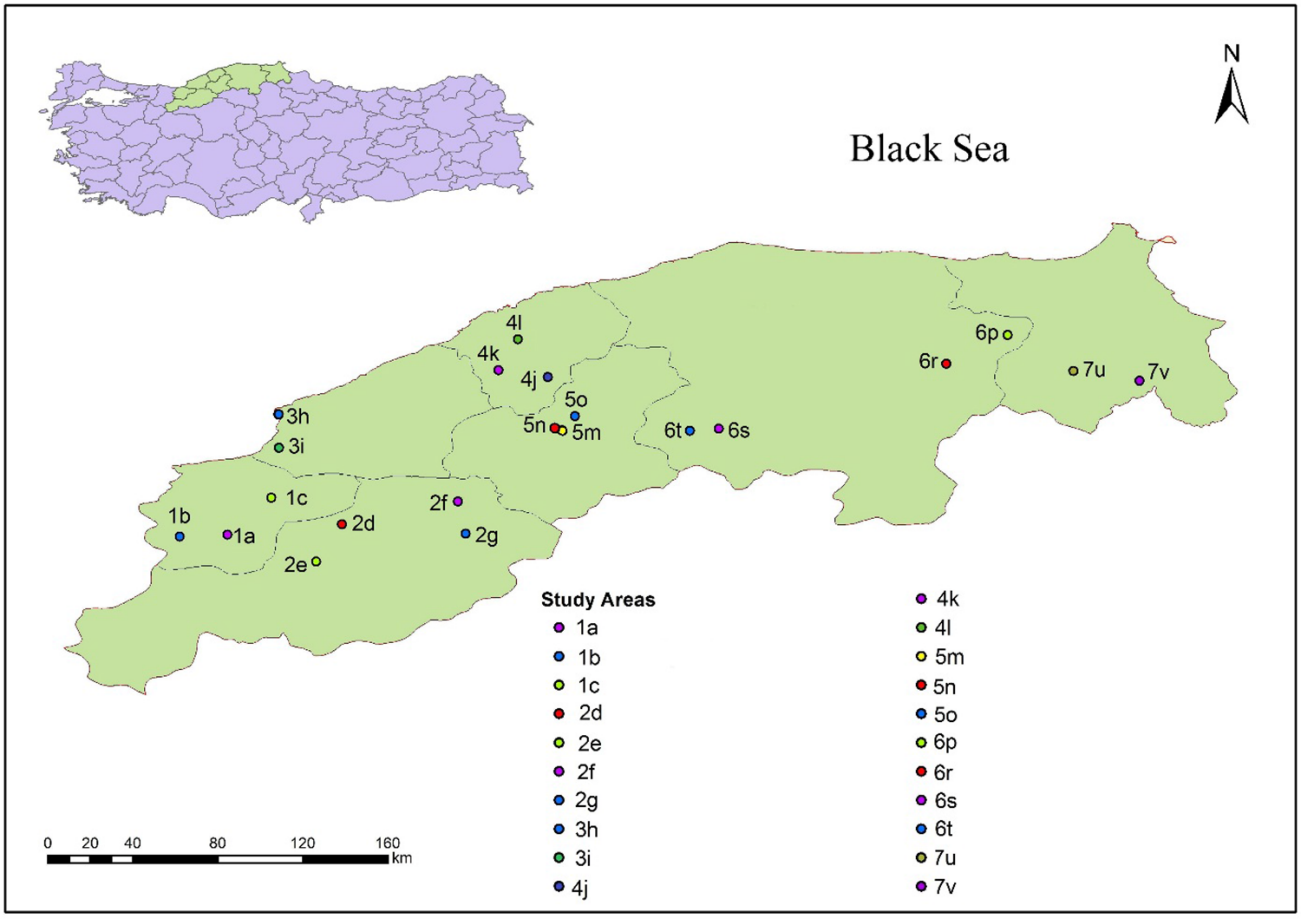
Map showing the log depots in the study areas (Provinces: 1-Duzce (DU), 2- Bolu (BO), 3-Zonguldak (ZO), 4-Bartın (BR), 5-Kastamonu (KS), 6-Karabuk (KR) and 7-Sinop (SI). Letters on the map are the codes of the log depots in the provinces of the study area. The map was plotted using by ArcGis 10.3 version which is used for mapping and editing tasks).

The map in Fig. 1 shows the 21 log storage facilities of the seven provinces covered in the study, including Duzce (DU), Bolu (BO), Zonguldak (ZO), Bartın (BR), Kastamonu (KS), Karabuk (KR) and Sinop (SI) in Northwest Turkey.

The current study took place in these 21 log depots in provinces located in the Western Black Sea Region of Turkey. The log depots were selected on the basis of the amount of stored wood and number of different wood species. The highest numbers of wood species were identified in log depots 1a and 1b. Table 1 presents details of the log depots such as their aspect, altitude and distance from forest areas.

**Table 1:** Log depot details: aspect, altitude and distance from forest areas.

Log Depot Code	Aspect	Altitude (m)	Distance to forest (m)	Forest type at depot site
1a	E	199	> 3,000	–
1b	NE	129	> 3,000	–
1c	NW	289	< 3,000	Deciduous
2d	S	1,166	< 3,000	Coniferous
2e	NE	794	< 3,000	Coniferous
2f.	NW	690	< 3,000	Coniferous
2 g	S	1,270	> 3,000	–
3 h	SW	39	> 3,000	–
3i	S	19	< 3,000	Deciduous
4j	N	506	< 3,000	Deciduous
4 k	SW	538	> 3,000	–
4 l	SW	785	> 3,000	–
5 m	SW	308	< 3,000	Coniferous
5n	SW	71	< 3,000	Coniferous
5o	NE	35	< 3,000	Coniferous
6p	W	431	> 3,000	–
6r	W	657	> 3,000	–
6 s	F	603	< 3,000	Coniferous
6t	NE	604	< 3,000	Coniferous
7u	F	342	> 3,000	–
7v	F	277	< 3,000	Coniferous

E: East, W: West, N: North, S: South, F: Flat.

### Installation of insect collection cages in log depots

At the beginning of the April 2015 and 2016, a single insect collection cage was installed in each of the 21 log depot facilities. The cages were then collected at the end of October of the mentioned years. Each cage was equipped with a data logger to measure the temperature and relative humidity within the cage environment. During the study, monthly average temperature and relative humidity values at each cage location were recorded. These data were later correlated with insect frequency, species number and insect density of the Cerambycidae and Buprestidae families. Furthermore, a mixture of trans-verbenol (100 mg) + myrtenol (100 mg) + alpha pinene (20 mg), Ipsdienol (140 mg), Ipsenol, 2-methyl 3-butene 2-ol and cis-verbenol was used as a pheromone entrapment agent. The cages were newly developed^24^ and had some features that differed from the standard Scandinavian insect collecting cages.

**Figure 2:**
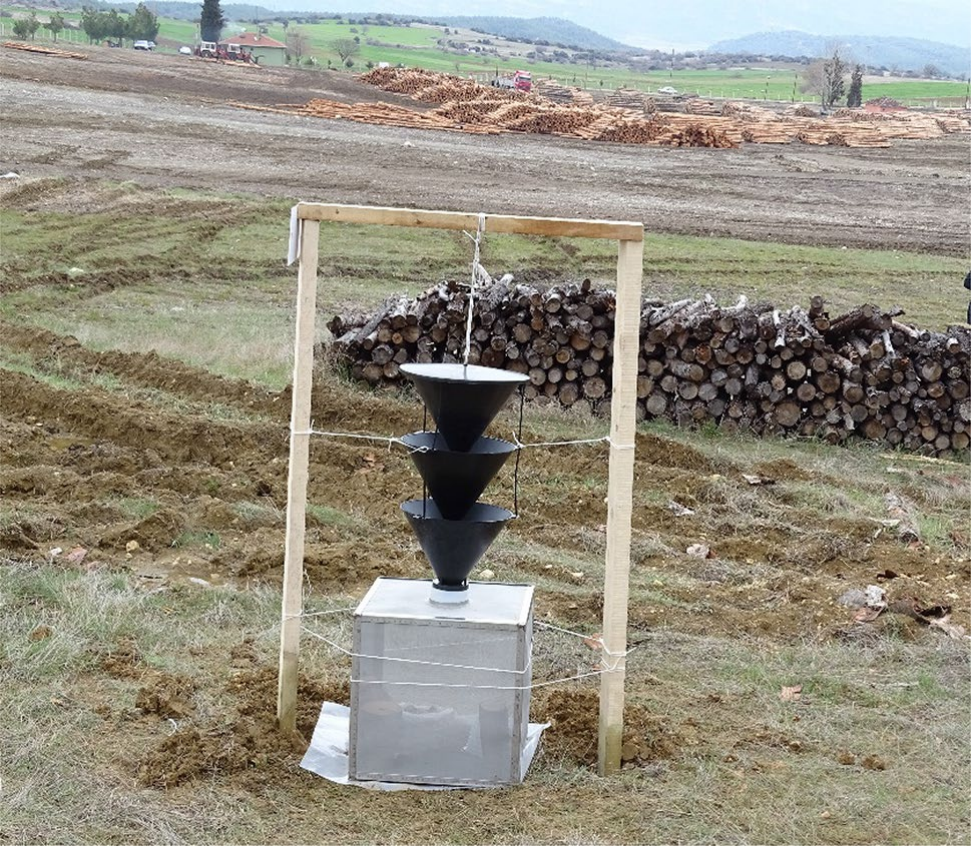
Insect collecting cage system integrated into Scandinavian type three funnel traps.

In production, the stainless steel gusset of the cages were cut to 50 × 50 × 50 cm and were joined in cubic way by spot welding. After this stage, stainless steel AISI 304 wires are cut according to gusset dimensions and were connected to gussets by using spot welding. The neck region where the collection containers were placed was connected on the welding machine. Cage covers were designed and manufactured to fit the frame system on a separate line. Finally, the cages were integrated to Scandinavian type three funnel trap system (Fig. 2).

These cages occupy a much larger area so that the entrapped insects can hide easily from their predators. As a result, the entrapped insects could be found live with body shape intact during the inspections.

Furthermore, some stored wood within the log depots was checked and split into small pieces and insects that damage wood were collected.

### Collection and identification of insects

The cage locations were visited at the end of the every month and the entrapped insects were collected. At the same time, the pheromone entrapment chemicals were refreshed. The collected insects were transferred to plastic containers with 10 air holes on their lids. Each plastic container was marked with collection date, number of insects and the location code. The identifications were made under a laboratory stereoscopic microscope^14,16,25–32^.

### Bevan damage severity index calculation

After the insect species identification was performed, the insects were separated into five groups based on the scale of their economic damage to wood (Table 2). The Bevan damage classification index^24^ was originally designed for living trees, but in the current study it was used to classify damages on harvested wood material.

**Table 2:** Insect damage scale values according to damage type.

Insect damage scale value	Damage type
0	Predator insects of bark insects; feed on decay fungi on wood
1	Insects not directly damaging wood material but breeding between bark and wood; cause minor economic losses
2	Insect damaging wood surface only and insects harming decaying wood and their predators
3	Insects causing heavy damage to wood
4	Insects generating excessive damage to wood material; lower technical specifications and cause major economic losses

The Bevan damage index can be calculated based on the damage scale and the frequency of insects found for a particular location. The Bevan damage index was calculated for each species based on the two-year field data, observations and previous literature studies. This classification is not based on insect population density, but on their tendency for wood damage. The damage of the insects was calculated using the following formula.

1 $$Damage\,index =\frac{Insect\,frequency\,\times\,Damage\,scale\,value}{Total\,damage\,index\,of\,the\,insect\,species} \times 100$$

Insect frequency is the number of appearances of insect species in the region at difference time. Damage scale value is a value according to damage type based on previous literature studies (Table 2). Bevan damage index was calculated with multiplication of insect frequency and damage scale value.

### Relationship of the Species of Cerambycidae and Buprestidae with environmental factors

The species of Cerambycidae and Buprestidae families are particularly important since they consume wood material as food, causing economic losses and having high damage index. They can damage a large variety of material, from newly harvested to partially decayed wood. To identify the optimum timing for insect management during a calendar year, insect frequency, number of species and insect density of the species of Cerambycidae and Buprestidae families and environmental data (temperatures and relative humidity) were recorded for each month. The insect density was calculated as;

$$D=\frac{n/a}{t}$$

where D: Insect density, n: insect number, a: field, t: time.

### Statistical analyses

Statistical analyses were conducted using SPSS 19 software. A Pearson correlation test was used to determine the correlation of the insect frequency, species number and insect density with the monthly average temperature and relative humidity.

## Results

A total of 7,312 insects were collected and identified from the pheromone traps and 1,022 insects from trapped on stored logs and wood in the all forest depots in this study. Species belong to twenty-four families were identified in the pheromone traps and stored wood. These families consisted of 94 genera and 124 different species. The Cerambycidae were represented by 35 different species, the highest among all the families.

### Bevan Damage Indices of insects found in the study areas

Table 3 shows the Bevan damage indices of insect species identified in the current study. The highest damage index values of insects collected from the pheromone traps were recorded as *Arhopalus rusticus* (9%), *Monochamus galloprovincialis* (8.5%), *Ips sexdentatus* (7.2%), *Acanthocinus aedilis* (6.9%), *Rhagium inquisitor (*6.8%), *Acanthocinus griseus* (6.2%), *Buprestis dalmatina* (5.1%), *Lacon punctatus* (5%)*, Buprestis haemorrhoidalis* (3.6%), *Camponotus vagus* (3.3%), *Hylotrupes bajulus* (3%), *Spondylis buprestoides* (2.9%), and *Buprestis octoguttata* (2.8%).

**Table 3:** Bevan damage indices of identified insect species.

Family	Insect species	Provinces (%)							P (%)	S-T (%)
		DU	BO	ZO	BR	KS	KR	SI		
Cerambycidae	*Anastrangalia reyi*			5.8					0.2	
	*Hylotrupes bajulus*	4.9	3.8	7.7	6.0	1.5	1.9	1.6	3.0	13.2
	*Chlorophorus herbstii*	0.8							0.1	
	*Chlorophorus varius*	3.6	1.4		1.5	2.9			1.6	
	*Chlorophorus sartor*						1.2		0.2	
	*Clytus arietis*									1.2
	*Ropalopus clavipes*				1.5				0.2	
	*Cerambyx scopolii*	1.6	0.9						0.5	
	*Purpuricenus budensis*					1.0			0.2	
	*Isotomus speciosus*					1.9	1.2	1.2	0.7	
	*Plagionotus arcuatus*	1.6	0.9						0.5	
	*Xylotrechus arvicola*				3.0				0.3	
	*Xylotrechus antilope*				1.5				0.2	
	*Xylotrechus rusticus*	1.2	0.7		1.5				0.5	
	*Phymatodes testaceus*	1.6	3.3		2.0	1.9	0.8	0.8	1.8	1.3
	*Leptura quadrifasciata*			5.8					0.2	1.0
	*Leptura aurulenta*									1.0
	*Stictoleptura cordigera*	1.2							0.2	
	*Rhagium bifasciatum*	1.6	1.9			1.3	3.3		1.4	
	*Rhagium inquisitor*	6.5	6.1	23.1	8.0	3.9	11.7	3.2	6.8	4.0
	*Stictoleptura scutellata*	1.2							0.2	2.0
	*Anastrangalia sanguinolenta*					1.0	1.2		0.3	1.0
	*Leiopus femoratus*							0.8	0.1	
	*Monochamus galloprovincialis*	8.5	8.5		7.5	3.8	17.4	8.3	8.5	2.0
	*Acanthocinus aedilis*		9.2		6.0	13.4	3.7	7.1	6.9	
	*Acanthocinus griseus*	4.9	7.8	0.0	6.0	6.7	6.2	6.0	6.2	
	*Prionus coriarius*			5.8				1.2	0.3	
	*Oxypleurus nodieri*					0.6			0.1	
	*Anisarthron barbipes*		0.5						0.2	
	*Asemum striatum*	2.4	3.5	0.0	1.5	1.9	1.2	1.2	2.1	
	*Arhopalus ferus*						1.2		0.2	
	*Arhopalus rusticus*	1.6	6.6	23.1	10.0	14.0	8.3	11.1	9.0	4.0
	*Spondylis buprestoides*	1.2	6.4		6.0	1.0	1.2	1.2	2.9	
	*Ergates faber*									1.3
	*Aegosoma scabricorne*									1.3
Buprestidae	*Anthaxia semicuprea*	0.4							0.1	
	*Chrysobothris affinis*					0.3	0.4	0.4	0.2	
	*Chalcophora detrita*					1.9		8.7	1.6	0.7
	*Chalcophora mariana*		0.7			2.9	2.5	2.4	1.6	1.0
	*Agrilus aurichalceus*						0.8		0.1	
	*Dicerca berolinensis*	1.2	0.7						0.3	
	*Dicerca chlorostigma*	1.2							0.2	
	*Buprestis novemmaculata*		1.9			1.3			0.7	
	*Buprestis humeralis*						2.5		0.3	
	*Buprestis octoguttata*		4.3			4.8	1.2	4.8	2.8	
	*Buprestis octopunctata*									1.0
	*Buprestis dalmatina*					5.7	11.2	17.3	5.1	1.0
	*Buprestis haemorrhoidalis*		7.6			1.3	5.0	5.4	3.6	
	*Perotis lugubris*							0.4	0.1	
	*Acmaeodera ottomana*								0.1	
Curculionidae/Scolytinae	*Tomicus minor*	0.4							0.1	
	*Tomicus piniperda*		0.2			0.3			0.1	
	*Orthotomicus erosus*		0.9		1.0			1.6	0.6	0.3
	*Orthotomicus proximus*	0.8	0.5						0.2	
	*Xyloborus eurographus*						0.8		0.1	0.3
	*Xyleborus monographus*			5.8					0.2	
	*Ips acuminatus*							0.4	0.1	
	*Ips sexdentatus*	7.3	7.1	13.5	10.4	7.6	6.2	4.0	7.2	3.0
	*Taphrorychus villifrons*	0.4							0.1	
	*Dryocoetes autographus*	0.4							0.1	
Curculionidae	*Hylurgus ligniperda*	0.8			1.0			1.6	0.5	
	*Hylastes Attenuatus*	0.8							0.1	
	*Rhyncolus elongatus*	0.8							0.1	1.3
	*Rhyncolus ater*				2.0				0.2	3.3
	*Pityokteines curvidens*	1.6				0.3			0.3	0.3
	*Hylobius abietis*	0.8	1.4	3.2	2.0	0.6		0.4	0.9	
	*Pissodes pini*	0.4					0.4		0.1	
	*Pissodes piceae*	0.8	0.7	0.0	0.5	1.3	0.4		0.6	
	*Magdalis duplicata*								0	
Tenebrionidae	*Opatrum sabulosum*									0.7
	*Helops caeruleus*									0.3
	*Helops rossii*									0.7
	*Gonodera luperus*	0.9			0.5		0.8		0.3	
	*Uloma cypraea*	1.5							0.1	2.6
	*Uloma culinaris*			1.2		0.6			0.3	3.3
	*Menephilus cylindricus*			0.9		0.9			0.1	4.0
	*Corticeus fraxini*									0
	*Corticeus pini*								0	0
Elateridae	*Melanotus castanipes*								0	
	*Calais parreysii*								0	
	*Lacon punctatus*	11.3	1.9		3.0	5.7	4.1	6.3	5.0	1.3
	*Ampedus elegantulus*	0.4		2.4		0.3			0.4	0.7
	*Ampedus pomorum*				1.0				0.1	0.3
	*Ampedus nigroflavus*									0.3
	*Ampedus sanguineus*	0.8							0.1	
	*Synaptus filiformis*									0.3
Anobiidae	*Ptilinus fuscus*									2.6
	*Xestobium rufovillosum*	1.6							0.2	
	*Anobium punctatum*		1.9						0.5	5.3
	*Ernobius mollis*					0.3			0.1	
Scarabaeidae	*Trichius sexualis*							0.8	0.1	0.7
	*Cetonia aurata*	0.8	1.4		1.0	0.6		0.8	0.8	1.3
	*Amphimallon solstitiale*									0
	*Valgus hemipterus*									1.0
Melandryidae	*Serropalpus barbatus*		3.5		3.0	1.9			1.6	
	*Rushia parreyssi*									1.3
	*Melandrya dubia*	2.4							0.3	
Lucanidae	*Sinodendron cylindricum*									0.7
	*Dorcus parallelipipedus*	10.5			3.0		0.8	0.8	2.1	12.5
Cossidae	*Zeuzera pyrina*									0.7
Siricidae	*Sirex noctilio*				1.5				0.2	
Rhinotermitidae	*Reticulitermes* sp									1.3
Formicidae	*Camponotus vagus*	6.1	2.1		7.5	3.8	2.5		3.3	7.9
Lycidae	*Lygiopterus sanguineus*									0.7
Cantharidae	*Cantharis fusca*	0	0						0	
	*Cantharis livida*	0							0	0
	*Cantharis rufa*		0.5						0.1	0.3
	*Cantharis rustica*	0							0	
Cleridae	*Trichodes apiarius*	0	0		0	0	0		0	0
	*Thanasimus mutillarius*		0			0	0	0	0	0
	*Thanasimus formicarius*	0	0	0	0	0	0	0	0	0
Trogositidae	*Tenebroides fuscus*	0							0	
	*Temnochila caerulea*				0	0	0	0	0	0
	*Ostoma ferruginea*									0
	*Calitys scabra*								0	
Zopheridae	*Aulonium ruficorne*	0	0		0	0	0	0	0	
	*Bitoma crenata*				0.5				0.1	
	*Pycnomerus sulcicollis*	0.4		1.9				0.4	0.2	0.3
Dasytidae	*Aplocnemus alpestris*									0
	*Tomoxia bucephala*	0.4	0.2		0.5				0.2	
Nitidulidae	*Glischrochilus hortensis*		0.2						0.1	
	*Rhysodes sulcatus*									0.7
Silvanidae	*Uleiota planata*									0.3
Oedemeridae	*Chrysanthia viridissima*		0.5						0.1	
Pentatomidae	*Apodiphus amygdali*					0			0	

P, Pheromone traps, S-T, Trapped on stored logs and wood.

The Elateridae and Tenebrionidae families, on the other hand, were more numerous in species, but exhibited lower damage index values when compared to the other species. Elateridae and Tenebrionidae larvae feed on larvae of other wood-destroying insects, which limits their damage to wood material. A few exceptions were observed and recorded on the stored wood. These included the *Uloma culinaris, Uloma cyprea* and *Lacon punctatus* species, which made tunnels in the long-term stored wood, resulting in higher damage index values compared to the damage index values of the pheromone-trapped insects.

Furthermore, Bevan damage index values were found to be highly dependent on the location of the pheromone traps. *Arhopalus rusticus,* for example, resulted in a 9% damage index value for the Western Black Sea Region. When each province was taken into consideration its damage indices varied from 1.6% for Duzce to 23.1% for Zonguldak. In these results, variation of wood species may play an important role in the frequency of insect species. In addition, the presence of wood species appropriate for the insects and climatic conditions contributed to the damage index values.

Table 3 indicates the Bevan damage index values of larvae, pupae and adult insects found in traps and in stored wood in log depots in the Western Black Sea Region. The highest damage index values were identified as 13.2%, 12.5%, 7.9%, 5.3% and 4% for *H. bajulus, Dorcus parallelipipedus*, *C. vagus, Anobium punctatum*, and *A. rusticus,* respectively*. Hylotrupes bajulus* causes damage on softwoods, hardwoods and freshly sawn construction wood. *Hylotrupes bajulus* and *A. punctatum* are considered the two leading insect species causing major economic losses in wood and wood products.

Table 4 relates the frequency of species to the distance of depots to forest areas. The data indicated that the frequency of *M. galloprovinciallis* was 75.6% for depots closer to forest land, while it was only 24.4% for log depots further from forests. Furthermore, insect density also showed similar trends of higher values for depots closer to forest land. The frequency of insects damaging living trees was also recorded as higher in locations closer to forest land (Table 4).

**Table 4:** The frequency, and insect density depending on distances to forested areas.

Insect species	Storage location	Insect frequency		Insect density number/ha*year
		(f)	(%)	
*Monochamus galloprovincialis*	Further to forest than 3 km	12	24.4	2,2
	Closer to forest than 3 km	37	75.6	6,9
*Buprestis dalmatina*	Further to forest than 3 km	8	27.5	1,7
	Closer to forest than 3 km	21	72,5	7,5
*Spondylis buprestoides*	Further to forest than 3 km	2	11.7	0,1
	Closer to forest than 3 km	15	88.3	3,7

### Correlation of environmental factors with Cerambycidae and Buprestidae

Table 5 shows the relation between insect species of Cerambycidae and mean monthly temperatures, relative humidity, insect frequency, insect density, and number of species. When the environmental factors were considered, insect frequency, insect density and number of species were positively correlated at a moderate level, however, this was not significant (*p* > 0.05). There was a strong and significant positive relationship between insect frequency, insect density and species number (*p* < 0.05). On the other hand, a strong and significant positive correlation was found between the number of species and insect density (*p* < 0.05, r: 0.945).

**Table 5:** Correlation between the species of Cerambycidae family and environmental factors.

Correlation source	Mean monthly temperature	Mean monthly RH	Insect frequency	Species number	Insect density
**Mean monthly temperature**					
r	1				
*p*					
**Mean monthly RH**					
r	− 0.544	1			
*p*	0.206				
**Insect frequency**					
r	0.502	0.291	1		
*p*	0.251	0.527			
**Species number**					
r	0.481	0.261	0.982**	1	
*p*	0.274	0.572	0.000		
**Insect density**					
r	0.454	0.361	0.985**	0.945**	1
*p*	0.306	0.426	0.000	0.001	

** Correlation is significant at 0.01. r: Correlation coefficient *p*: Significance level, r: 0.00 No correlation, 0.01–0.29 Low-level correlation, 0.30–0.70 Intermediate-level correlation 0.71–0.99 High-level correlation, 1.00 Excellent level of correlation.

Table 6 highlights a very strong positive correlation of monthly mean temperature with insect frequency, number of species and insect density for the Buprestidae (*p* < 0.005, r_1_: 0.922, r_2_: 0.879, r_3_: 0.942).

**Table 6:** Correlation between the species of Buprestidae family and environmental factors.

Correlation source	Mean monthly temperature	Mean monthly RH	Insect frequency	Species number	Insect density
**Mean monthly temperature**					
r	1				
*p*					
**Mean monthly RH**					
r	− 0.544	1			
*p*	0.206				
**Insect frequency**					
r	0.922**	− 0.511	1		
*p*	0.003	0.241			
**Species number**					
r	0.879**	− 0.197	0.912**	1	
*p*	0.009	0.672	0.002		
**Insect density**					
r	0.942**	− 0.517	0.981**	0.880**	1
*p*	0.002	0.235	0.000	0.004	

**Correlation is significant at 0.01. r: Correlation coefficient *p*: Significance level. r: 0.00 No correlation. 0.01–0.29 Low-level correlation. 0.30–0.70 Intermediate-level correlation 0.71–0.99 High-level correlation. 1.00 Excellent level of correlation.

## Discussion

It can be easily seen that insects from the Cerambycidae and Buprestidae families exhibited the highest damage index values compared to the damage index values of the other families. This could be explained by the fact that the saproxylic features of the larvae belong to Cerambycidae enable them to feed on living trees and wood material^10,33^. The larvae belong to Buprestidae family also consumes living trees, felled trees and stumps^34^.

The current study also indicated that *Ips sexdentatus* of the Scolytinae (Curculionidae) family rated high on the damage index due to their higher frequency in the field and higher breeding capacity^35^. These insects damage the cambium layer between the wood and bark causing excessive tree deaths^36^.

Cleridae, Trogossitidae and other predator insect families such as Cantharidae, Zopheridae and Dasytidae resulted in zero Bevan damage indices. This could have been a result of the larvae of these groups feeding on larvae of wood-destroying insects, which limited their ability to damage wood^4–6^.

The Mordellidae, Cerylonidae and Nitidulidae families displayed lower Bevan damage index values since insects belonging those families feed on wood-decaying fungi and tree sap^37–39^.

On the other hand, *A. griseus* exhibited a damage index value of 4.9–7.8% regardless of location in the region, indicating a homogeneous distribution^40^.

The findings demonstrated that each insect species could give different damage index values depending on whether they were found on stored logs or in pheromone traps. For example, *M. galloprovinciallis* showed an 8.5% damage index value among the pheromone-trapped species. The damage index was reduced to 2% among those species found in wood of stored logs. This could be because some log storage areas are closer to forest land than others. Thus, *M. galloprovinciallis* could have had easy access to dead or weak trees^16,22,41^.

Some insects captured by pheromone traps showed much lower damage index values than their damage index values on long-term stored wood. *Hylotrupes bajulus* and *A. punctatum*, for example, had very high damage indices on long-term stored wood. A possible explanation of why these species showed a lower damage index in pheromone traps could be the utilization of a general attracting pheromone. However, it is known that *H. bajulus* can be attracted by a species-specific pheromone called (3R)-3-hydroxy-2-hexanone^42^. Another species-specific pheromone is 2,3-dihydro-2,3,5-trimethyl-6-(1-methyl-2-oxobutyl)-4H-pyran-4-one (stegobinone) for *A. punctatum*^43^. The current study, however, did not used species-specific pheromones. On the other hand, it was thought that *H. bajulus* rarely fly at temperatures under 25–30 °C and this was believed to be effective on the results^44^.

It is well known that temperature has positive effects on insects. However, some Cerambycidae species did not show a significant correlation with mean monthly temperature and relative humidity because the periods differed greatly on a monthly basis. Most insects of the Cerambycidae family reached maturity in June and July. *Acanthocinus aedilis* matured before the summer months. Some species, e.g., *Prionus coriarius*, matured around the end of August and September. Species belong to *Rhagium* and *Spondyses* developed into the same stage after May or June in Europe site^45,46^.

The relationship between the number of species and insect density of this family might be explained by their ability to lay a high number of eggs. *Ergates faber,* for example, can lay up to 320 eggs^47^.

**Figure 3:**
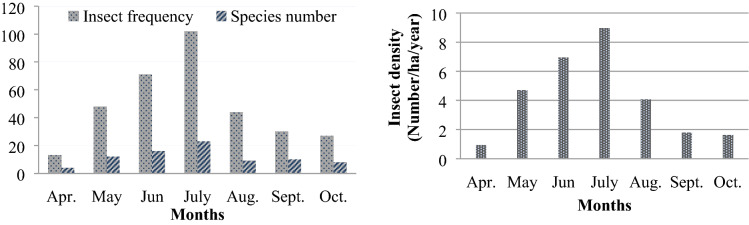
Total insect frequency, species number and insect density changes of the species of Cerambycidae family depending on months for 2015–2016 years.

Figure 3 displays the monthly insect frequency, number of species and insect density values of the Cerambycidae family throughout the 2-years observation period. It is clearly shown that the insect frequency and insect number values reached their peak in July. Therefore, the summer months can be considered the best time for management against the species of this family. A similar phenomenon was recorded for Buprestidae insects, but their peak time was recorded in August instead of July. This can be explained by their higher temperature requirements compared to the Cerambycidae^48,49^. Some members of this family are known as heliophilus^50^.

**Figure 4:**
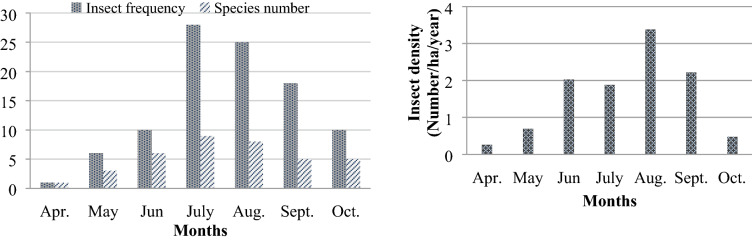
Total insect frequency, species number and insect density changes of Buprestidae family insects depending on months for 2015–2016 years.

Figure 4 displays monthly changes in insect frequency, species number and insect density values throughout the observation period. It is evident that insect frequency and number of Buprestidae insect species peaked in July, while insect density was at its maximum in August.

## Conclusion

The severity of destruction of wood-destroying insects found in log depots and stored wood material was calculated according to the Bevan damage index. The Cerambycidae and Buprestidae insects found in the pheromone traps exhibited the highest damage index values.

The most harmful species on long-term stored wood were listed as *H. bajulus* with 13.2%, *D. parallelipipedus* with 12.5% and *C. vagus* with 7.9% damage values.

The lowest damage index values were found for predators of bark insects (Cleridae, Trogossitidae) and insects that feed on wood-colonizing fungi such as those of the Mordellidae, Cerylonidae and Nitidulidae families.

Some Elateridae and Tenebrionidae species displayed relatively high damage values on long-term stored wood. These species lower the technical specifications of wood by tunneling.

Cerambycidae and Buprestidae insects displayed a positive correlation of insect frequency, number of species and insect density values with environmental factors (temperature and RH) during the summer months of July and August. Therefore, the most effective time for management measures against these insects should be that time of the year.

With this study, insect species that can cause damage wood in outdoor storage in Western Black Sea region have been evaluated according to Bevan damage index and the importance of insects in terms of the damage wood in the forest industry sector have been revealed. Thus, a basis was created for the fighting with harmful insects.

## Data Availability

Availability of materials and data The datasets generated and/or analyzed during the current study are available in the Zenodo repository (Yalcin et al. 2019). Datasets not peer-reviewed. Mesut Yalcin, Caglar Akcay, Besir Yuksel, Cihat Tascioglu, & Ali Kemal Ozbayram. (2019). Determination of the Damage Severity of Wood-Boring Beetles According to the Bevan Damage Classification System [Data set]. Zenodo. https://doi.org/10.5281/zenodo.2788797
